# Processing speed training increases the efficiency of attentional resource allocation in young adults

**DOI:** 10.3389/fnhum.2013.00684

**Published:** 2013-10-18

**Authors:** Wesley K. Burge, Lesley A. Ross, Franklin R. Amthor, William G. Mitchell, Alexander Zotov, Kristina M. Visscher

**Affiliations:** ^1^Department of Psychology, Roybal Center for Research on Applied Gerontology, University of Alabama at BirminghamBirmingham, AL, USA; ^2^Department of Human Development and Family Studies, The Pennsylvania State UniversityState College, PA, USA; ^3^Department of Psychology, University of Alabama at BirminghamBirmingham, AL, USA; ^4^Department of Vision Science, Vision Science Research Center, University of Alabama at BirminghamBirmingham, AL, USA; ^5^Department of Neurobiology, University of Alabama at BirminghamBirmingham, AL, USA

**Keywords:** cognitive training, pupillometry, attentional resources, cognitive intervention, UFOV

## Abstract

Cognitive training has been shown to improve performance on a range of tasks. However, the mechanisms underlying these improvements are still unclear. Given the wide range of transfer effects, it is likely that these effects are due to a factor common to a wide range of tasks. One such factor is a participant's efficiency in allocating limited cognitive resources. The impact of a cognitive training program, Processing Speed Training (PST), on the allocation of resources to a set of visual tasks was measured using pupillometry in 10 young adults as compared to a control group of a 10 young adults (*n* = 20). PST is a well-studied computerized training program that involves identifying simultaneously presented central and peripheral stimuli. As training progresses, the task becomes increasingly more difficult, by including peripheral distracting stimuli and decreasing the duration of stimulus presentation. Analysis of baseline data confirmed that pupil diameter reflected cognitive effort. After training, participants randomized to PST used fewer attentional resources to perform complex visual tasks as compared to the control group. These pupil diameter data indicated that PST appears to increase the efficiency of attentional resource allocation. Increases in cognitive efficiency have been hypothesized to underlie improvements following experience with action video games, and improved cognitive efficiency has been hypothesized to underlie the benefits of PST in older adults. These data reveal that these training schemes may share a common underlying mechanism of increasing cognitive efficiency in younger adults.

## Introduction

Attention is a limited cognitive resource (Sigman and Dehaene, 2006) that is important for everyday functioning. People perform better on attention demanding tasks when more attentional resources are available to devote to those tasks (Duncan et al., 1994). Thus, developing ways to improve people's efficiency using these attentional resources is essential in order to free more resources and improve performance of attention demanding tasks.

Cognitive abilities are modifiable through targeted cognitive interventions and action-based video games (Ball et al., 2002; Green and Bavelier, 2006). Importantly, improvements resulting from some of these targeted interventions have been found to transfer to other tasks such as driving and measures of processing speed (Ball et al., 2002; Green and Bavelier, 2006). The mechanisms underlying these improvements in cognitive abilities are not fully understood. Given that training transfers to a wide breadth of tasks, the underlying mechanism may be a basic one that is integral to many functions of daily living. The ability to efficiently allocate attention is important for a wide range of tasks and is therefore a good candidate mechanism that may underlie some of the broad improvements observed after training.

Previous research examining action video games and players as a cognitive training paradigm have shown that experience with these games impacts the attention system through improved selective attention, improved spatial attention, reduced costs of task switching, and increased visual processing speed (Green and Bavelier, 2003, 2006; Green et al., 2012). A different targeted cognitive training paradigm, processing speed training (PST) has been shown in randomized controlled trials to transfer to many diverse everyday activities including improved driving safety, activities of daily living, and health-related quality of life (Edwards et al., 2005b; Wolinsky et al., 2006; Ball et al., 2010) in older adults. PST has been shown to increase visual processing speed, as measured by the Useful Field of View (UFOV^1^) test (Ball et al., 2007). The UFOV test assesses the duration (ms) that increasingly complex stimuli must remain present in order to be interpreted (Edwards et al., 2005a, 2006). PST involves identifying simultaneously presented central and peripheral stimuli. As training progresses, the task becomes increasingly more difficult, by including the presence of peripheral distracting stimuli and decreasing the duration of stimulus presentation. Although PST translates to improved everyday function in older adults, the mechanism or mechanisms behind this transfer are unknown.

The purpose of the current study is to assess whether improved attentional resource allocation is one potential underlying mechanism of PST. The current study is novel as it uses pupillometry to examine how a modified version of PST impacts the allocation of attentional resources in a sample of young adults. Several studies have linked increased pupil diameter to increased cognitive effort, or attentional resource allocation. For example, larger pupil diameter is positively correlated with the increased difficulty of mental calculation (Hess and Polt, 1964) and with increased short-term memory load (Kahneman and Beatty, 1966). Additionally larger pupil diameter at encoding predicts that a participant will later remember an item (Papesh et al., 2012). Prior work has also shown that different aspects of attention have different effects on the pupil. In tasks that require more focused attention pupil diameter decreases. When the task requires more broad attention, pupil diameter widens (Daniels et al., 2012). Research has also shown that changes in pupil diameter (in the absence of luminance changes) reflect attentional resource allocation (Verney et al., 2004). For the purposes of this study, the operational definitions of the phrases, “cognitive effort,” “mental effort,” and “processing load” are interchangeable and will be referred to using the general term “use of attentional resources.”

The literature summarized above shows that larger pupil diameter during equiluminance is a measure of increased attentional resource use. Thus, monitoring changes in pupil diameter across tasks is an efficient method for assessing how attentional resource allocation changes as a function of training. The current study used this method to examine how a modified version of PST impacts allocation of attentional resources in a sample of young adults. We hypothesized that training would result in more efficient use of cognitive resources during demanding tasks. To the authors' knowledge, this is the first study to investigate the mechanisms of PST through pupillometry.

## Method

### Participants

Twenty young adults (mean age 23 years; range 19–31 years) were randomized to receive training (*n* = 10) or to the no-contact control condition (*n* = 10) and gave their written consent before the experiments. The institutional review board of the University of Alabama at Birmingham approved the experiment. Table 1 details the demographics of the sample used in this study. Inclusion criteria for this study were: no history of neurological disorders, not currently taking any psychoactive medication, and having normal or corrected-to-normal vision (20/40 or better).

**Table 1:** **Demographics**.

**Groups**	**Age**	**Gender**	**Race: AA**	**Race: caucasian**	**Race: other**
Trained	23.2 (19–28)	60% Female	2	4	4
Controls	22.9 (19–31)	60% Female	1	6	3

Race χ^2^ (2, n = 20) = 0.88, p = 0.64.

### Materials and procedure

#### Modified useful field of view test

Participants performed a modified version of the UFOV test with four levels of difficulty at both baseline and posttest. The computerized test was created in MATLAB (The MathWorks, Natick, MA), using the Psychtoolbox (Kleiner et al., 2007). Participants' heads were stabilized with eyes 36.61 inches from an 18-inch NEC Accusync 95F monitor. Screen resolution was 1024 × 768 with a refresh rate of 85 Hz. Responses were collected using a mouse, a method that has been shown to be reliable and valid for use with the standardized UFOV test (Edwards et al., 2005b).

**Figure 1:**
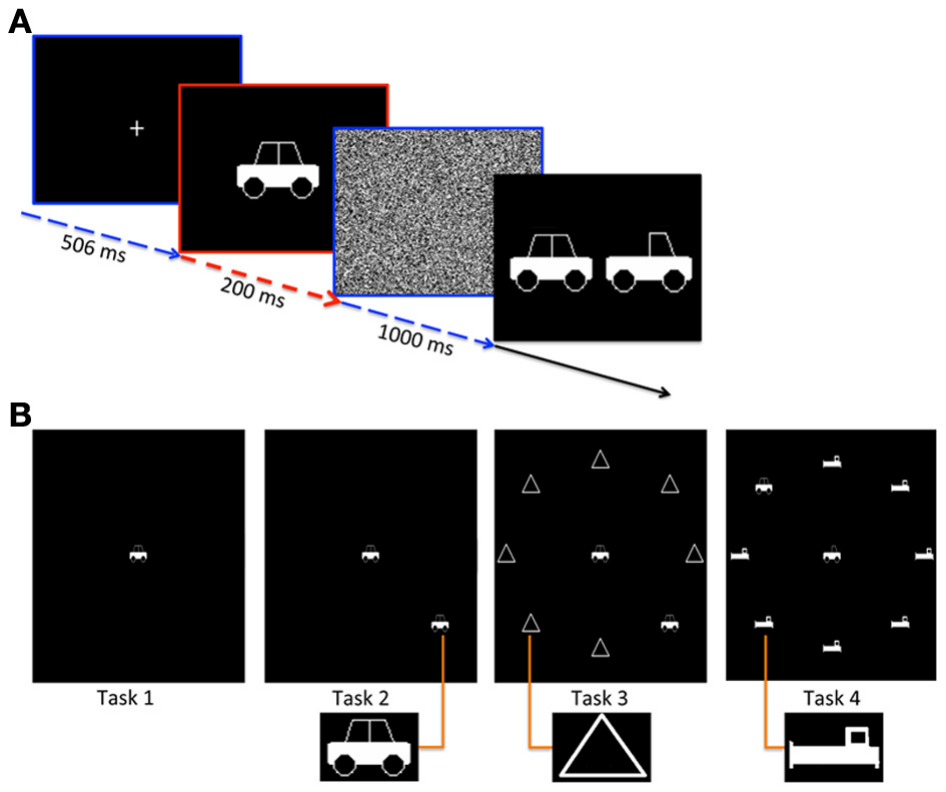
**(A)** Task timing for each trial of the test. A fixation cross was presented for 506 ms followed by the presentation of the stimulus for 200ms. Then a white noise mask was presented for 1000 ms followed by a memory probe. **(B)** Different task levels. Stimulus screen is shown for each of the 4 tasks. Task 1 includes a central stimulus with no peripheral stimuli. Task 2 includes the central stimulus plus a peripheral stimulus with no distractors. Task 3 adds triangle distractors and Task 4 includes distractors that appear more similar to the stimulus. Popouts show zoomed in images of the peripheral stimuli.

Figure 1 shows a schematic of one trial of the task during the easiest condition of the modified UFOV test (Task 1). Prior to each stimulus presentation, a fixation cross was presented for 506 ms to serve as a cue. This cue was followed by a centrally presented image of a car or truck for 200 ms. Immediately following the stimulus presentation, a full-screen white noise mask was presented for 1000 ms. Participants then indicated the identity of the central stimulus using the mouse to click a car or truck image, as seen in Figure 1A. Each stimulus subtended 3.15 degrees of visual angle. On fifty percent of trials the stimulus was a car; on the remainder it was a truck. Response time was not emphasized; participants were given as long as needed to respond to each trial.

The second task of the modified UFOV test (Task 2) was more challenging, as participants were asked to (1) identify the central stimulus (as in Task 1) as well as (2) localize a peripheral car stimulus that was presented simultaneously. The peripheral stimulus was placed randomly in one of 8 locations spaced equally around the periphery of the screen 5.7 degrees from the center. Figure 1B shows an example stimulus presentation for each task. Timing for this task was identical to Task 1, but after indicating the identity of the central stimulus, participants were prompted to use the mouse to click one of 8 locations indicating where the peripheral stimulus had been presented. Task 3 was identical to Task 2, but with the addition of distracting peripheral stimuli. These triangle distracters can be easily distinguished from the target stimuli. Task 4 was identical to Task 3, but the distracters were more similar to the car stimulus making the discrimination between target stimuli and distracters more difficult (see Figure 1).

Four tasks were chosen that involve different levels of visual attention. Task 1 involves focused attention toward the location of the central stimulus, while Tasks 2, 3, and 4 involve broad (or divided) attention toward both the central and peripheral locations. Because broad attention has been shown to elicit larger pupil diameter than focused attention, Tasks 2, 3, and 4 are analyzed independently from Task 1 (Daniels et al., 2012). Tasks 2, 3, and 4 involve divided visual attention, because the participant must attend to both the central and peripheral locations simultaneously. Tasks 3, and 4 additionally involve selective attention, because the participant must suppress processing of irrelevant distractors. Task 4 involves a more challenging selective attention task than Task 3, as the stimuli are similar to the distractors in Task 4. Thus, Tasks 2 and 4 differ in whether the participant must suppress processing of irrelevant target-like distractors.

During the baseline and posttest, stimulus presentation was kept identical, with stimulus presentation times fixed at 200 ms. Task level was incremented every 25 trials, in the order Tasks 1, 2, 3, 4, 1, 2, 3, 4, 1, 2, etc. The test phase included 500 total trials (5 sets of 25 trials of each of the 4 task levels).

#### Modified processing speed training

After the baseline test, participants were randomly assigned to a training or a no-contact control group. Training involved practicing the same stimuli as testing. A staircase algorithm adapted the difficulty level to the performance of the participant. This adaptive strategy maintains a level of challenge throughout the training and reduces the likelihood of reaching plateau effects. At the start of the session, participants were presented with Task 1, with a stimulus presentation duration of 200 ms. Trials were made more difficult (shorter presentation duration) or easier (longer presentation duration) based on performance checks every fourth trial. Possible stimulus durations were: 306, 259, 200, 153, 106, 82, 59, 35, and 24 ms.

These performance checks counted responses to the first (central) and second (peripheral) portion of each trial independently. This means that for Tasks 2, 3, and 4 there were two responses per trial, each of which contributed an individual correct or incorrect response to the total. For example, a participant performing Task 2 has a possible 8 responses in 4 trials. If they get 6 of those correct, they have performed at 75% accuracy. At each performance check, if performance on the preceding four trials was greater than 75%, the task was made more difficult by shortening the stimulus duration. If the stimulus duration was already as short as possible, the task difficulty was increased to the next level. For the most difficult level (Task 4), task difficulty was not increased and continued to run at the shortest stimulus duration possible.

If the group of four trials was less than 37.5% correct (3 of 8 responses correct), the stimulus duration was increased (i.e., made easier). If the duration was already at its longest presentation time, the task difficulty was decreased. If performance was between 37.5% and 75% correct, the task was kept the same. By this staircase method of increasing and decreasing task difficulty, participants generally moved toward the most difficult version of the task over the course of training. By the conclusion of training, all participants were performing at the most difficult level of the task.

### Procedure

The experiment consisted of 3 phases: a baseline test, six training sessions (for those randomized to training) and a posttest. Each of the six training sessions consisted of two 45-min training blocks of 375 trials each, with a 10-min break between the blocks.

During baseline, training and posttest, participants' eye movements and pupil diameters were recorded using an Eyelink 1000 eye tracker (SR Research Ltd, Ottawa, Ontario, Canada). Eye data were collected in “gaze mode” at a rate of 1000 Hz. Pupil diameter was determined using Eyelink's “centroid” algorithm. Eye position data were calibrated and validated every 25 trials using a 9-point calibration method with randomized target order. Pupil diameter is measured in arbitrary units with noise levels corresponding to a resolution of 0.01 mm for a 5 mm pupil.

Kahneman and Beatty (1966) showed that changes in pupil diameter due to cognitive load are strongest at the time of a memory probe and response. Accordingly, we defined a window of interest where we expected to see the strongest effects as the 500 ms prior to probe presentation. This occurred simultaneously with the final 500 ms of the white noise mask (Figure 1). During this period, the luminance was identical across all conditions. Eye Link software (SR Research Ltd, Ottawa, Ontario, Canada) was used to identify fixations occurring during this window, and pupil diameter was calculated during each fixation.

Because eye movements can influence measured pupil diameter, only the diameter during fixations were included in analyses. Further, because the units of pupil diameter may differ from session to session and from participant to participant because of subtle changes in the relative locations of the eye and camera, inferences in this paper are based on percent changes in average pupil diameter between conditions within the same session. Calculating percent change between conditions effectively normalizes these between-session differences, and allows comparisons across sessions and across groups (Einhäuser et al., 2008). Mean pupil diameter for Task 2 was defined to be zero to allow for easier comparisons. This was chosen, as it is the only task with central and peripheral stimuli and no distractors.

## Results

Only Tasks 2, 3, and 4 were used in the analyses because Task 1 involves narrow, rather than broad attention, known to influence pupil diameter independently of cognitive load (Daniels et al., 2012). Previous literature on the effects of PST omit Task 1 in their analyses as this task has a strong ceiling effect (Ball et al., 2002) even in older adults. Behavioral and pupillary responses for both groups are shown in Table 2.

**Table 2:** **Mean values for behavioral and pupil data**.

		**Pupil diameter (% change from Task 2)**			**Percent correct**			**Reaction time (seconds)**		
		**Task 2**	**Task 3**	**Task 4**	**Task 2**	**Task 3**	**Task 4**	**Task 2**	**Task 3**	**Task 4**
Pre	Trained	0	0.11	2.74	87%	87%	82%	1.00	0.92	0.97
Post	Trained	0	−0.71	−0.06	92%	91%	94%	0.89	0.90	0.90
Pre	Control	0	−0.30	1.55	92%	93%	90%	0.95	0.96	0.97
Post	Control	0	0.08	2.86	91%	91%	88%	0.95	0.94	0.94

No significant differences between groups at baseline.

### Behavioral

Prior to training, participants performed the task effectively with a high overall accuracy for all task levels (82–87%). ANOVAs revealed that there were no significant baseline differences between the groups (trained vs. control) on reaction time or percent correct. This was consistent across Tasks 2, 3, and 4 at baseline.

Three-factor repeated measures ANOVAs were conducted to investigate the effect of Group (trained vs. control), Time (baseline vs. posttest), and Task (2, 3, and 4) on percent correct and reaction time. The reaction time ANOVA revealed a significant main effect of Time [*F*_(1, 18)_ = 10.68, *p* < 0.01]; however, main effects of Group and Task were not significant (*p* > 0.05). This indicates that there was not a significant effect of training on reaction time in this sample. The percent correct ANOVA revealed a significant main effect of Task [*F*_(2, 36)_ = 4.63, *p* < 0.02] and significant interactions of Task × Time [*F*_(2, 36)_ = 3.23, *p* < 0.05] and of Group × Task × Time [*F*_(2, 36)_ = 5.30, *p* < 0.01]. Other effects and interactions were not significant. *Post-hoc t*-tests showed that accuracy was significantly lower for Task 4 than Task 2 [*t*_(19)_ = 2.56, *p* < 0.02] and 3 [*t*_(19)_ = 2.64, *p* < 0.02]. Tasks 2 and 3 were not significantly different in accuracy (*p*s > 0.05). These data indicate that Task 4 is more difficult than Tasks 2 and 3. The mean percent correct for all tasks combined increased by 4.8% after training in the trained group; however, there was no significant interaction of Group × Time (*p* > 0.05), perhaps reflecting the fact that both groups performed very well (close to ceiling) even prior to training.

### Pupil diameter

**Figure 2:**
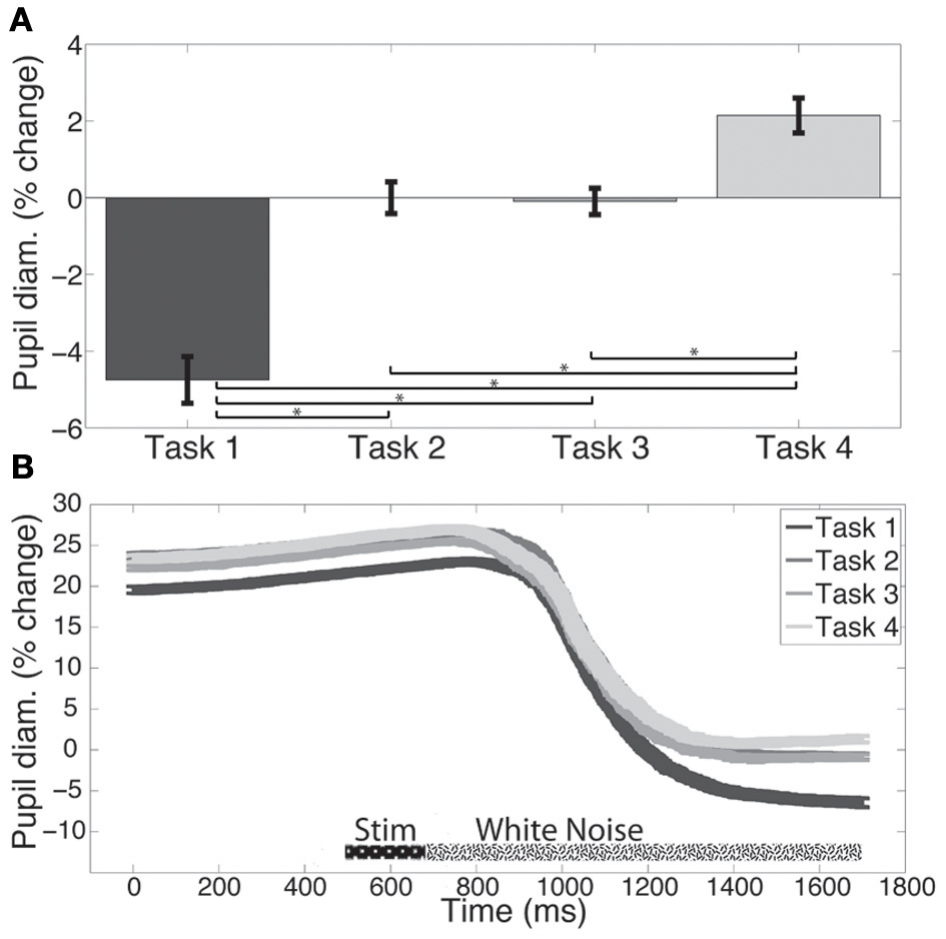
**(A)** Pupil diameter is larger for more difficult tasks. Pupil diameter units are % change from the Task 2 condition. Within-subject error bars are shown. **(B)** Task-evoked pupillary response across time. Control and training groups' baseline data representing the percent change of pupillary response over the different tasks. Values shown are percent change from the mean pupil diameter during the last 500 ms of the white noise for Task 2. Within subject error bars are shown. Data from the last 500 ms of the “White Noise” period correspond to the data in panel **2A**. ^*^p < 0.05.

Pupil diameter was measured to assess the attentional resources required by each of the tasks at baseline, during training, and at posttest. Figure 2A shows baseline data illustrating that pupil diameter during the memory maintenance period (500 ms prior to the probe) depends on task. Error bars show within-subject standard errors of the mean (Loftus and Masson, 1994; Cousineau, 2005) and are appropriate for comparing means for this within-subjects design. Note that the mean pupil diameter for Task 2 is defined to be 0 for easier comparisons between tasks, and to equate for individual participants' smaller or larger pupil diameters. Because the within-subjects error bars account for the variance across all the conditions, the error bars for Task 2 are not zero.

As with the behavioral data, ANOVAs were used to investigate if there were differences in the main variables of interest at baseline. Change in pupil diameter was investigated with a two factor ANOVA that included Group (trained vs. control) and Task (Tasks 2, 3, and 4) at baseline. There was a significant main effect of Task [*F*_(2, 36)_ = 8.82, *p* < 0.001]; however, there was no significant main effect of Group and no interaction indicating that baseline average pupil diameter was equal between the groups at baseline. *Post-hoc t*-tests showed that pupil diameter during Task 2 was significantly smaller than Task 4 [*t*_(19)_ = 3.01, *p* < 0.01], and that Task 3 was significantly smaller than during Task 4 [*t*_(19)_ = 3.74, *p* < 0.01]. These data indicate that pupil diameter is larger during more difficult tasks. There was no significant difference between Task 2 and Task 3.

In order to observe how pupil diameter changes dynamically throughout a trial, Figure 2B shows the normalized pupil diameter throughout all time periods of a trial including occasional breaks in fixation. The data are plotted as a percent change from a participant's pupil diameter during the memory maintenance period for Task 2. Thus, average pupil diameter during the 500 ms prior to the probe is defined as zero for the Task 2 condition to help visualize the differences between tasks. Within-subject error bars are provided in all figures.

A three-factor repeated measures ANOVA was conducted on percent change of pupil diameter from Task 2 with Group (trained vs. control), a Time (baseline vs. posttest), and Task (Tasks 2, 3, and 4). This ANOVA revealed a significant main effect of Task [*F*_(2, 36)_ = 14.88, *p* < 0.0001], a significant interactions of Group × Time [*F*_(1, 18)_ = 6.08, *p* < 0.025] and Group by Task × Time [F_(2, 36)_ = 3.35, *p* < 0.05]. This indicates that there was a significant effect of training on percent change in pupil diameter and significant difference between tasks as a function of training. Other effects and interactions were not significant at an alpha threshold of 0.05.

**Figure 3:**
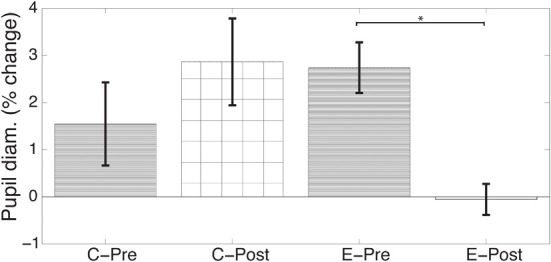
**Training abolishes the effect of task difficulty on pupil diameter**. Prior to training, both groups (Control, C; and Experiment, E) show increased pupil size during Task 4 relative to Task 2, consistent with an increase in task difficulty. After training, the experimental group shows no significant difference in pupil diameter between Tasks 2 and 4. Repeated measures analysis of variance shows this is a significant effect of training (interaction of Group by Time *p* < 0.03). ^*^p < 0.05.

Pupil diameter was measured for both participant groups during the baseline test and posttest to assess how attentional resource allocation was changed after training. Tasks 2 and 4 were chosen for comparison because both tasks involve responding to a central as well as a peripheral stimulus, keeping response demands identical. During the baseline, both the control and trained groups showed increased pupil diameter for the more difficult Task 4. This effect was not evident after training in the trained group (Figure 3). A two-factor repeated measures ANOVA on percent change of pupil diameter between Tasks 2 and 4 with Group (trained vs. control) and Time (baseline vs. posttest) showed an interaction between Group and Time [*F*_(1, 18)_ = 6.42, *p* < 0.03]. A follow up test to this interaction revealed that the trained group had a greater change in pupil modulation after training as compared to the control group [*t*_(18)_ = 2.309, *p* < 0.04]. Other effects and interactions were not significant at an alpha threshold of 0.05. This indicates that after training, the effect of task difficulty on pupil diameter is reduced.

**Figure 4:**
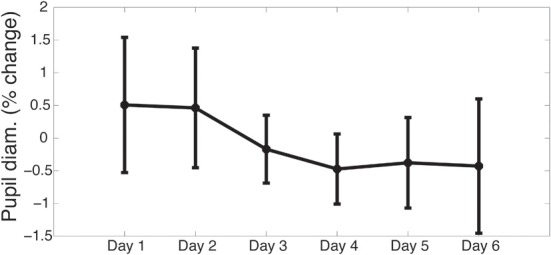
**Effect of task difficulty decreases throughout training**. Data from the first session of each training day are included here. No significant differences were observed (*p* > 0.05). However, the difference between tasks grows closer to 0 over time, consistent with the finding that pupil diameter during tasks of varying difficulties become more similar through training.

To further investigate this effect, pupil diameter during the training sessions was analyzed using the same method. Early training sessions showed larger differences in pupil diameter between Tasks 2 and 4 (Figure 4), consistent with Task 4 requiring significant attentional resources (baseline analysis). This difference decreased over training sessions, though not significantly (*p* > 0.05). This trend is consistent with the idea that training improves the efficiency of allocation of attentional resources, but no inference can be made based on these data as it is not statistically significant. The failure of this effect to reject the null hypothesis may be due to the fact that, because participants progressed through the training quickly, there were a relatively small number of trials completed during Task 2 as compared to Task 4, even as early as the first session of training. This was necessary to keep the training challenging, but decreased power. The trend in these data is consistent with the hypothesis that training causes a reduction in the effect of task difficulty on pupil diameter.

## Discussion

Our baseline results are consistent with previous research reporting that changes in pupil diameter reflect the cognitive effort required by a task (Beatty, 1982). Participants' pupil diameters increased with task difficulty, indicating that the more difficult processing speed tasks required more attentional resources. Pupil diameter was an excellent objective metric for assessing the effort required for a task.

Participants performed four tasks, which varied in difficulty because they involved different levels of visual attention. Task 1 involves focused attention toward the location of the central stimulus, while Tasks 2, 3, and 4 involve broad attention toward both the central and peripheral locations (Daniels et al., 2012). Tasks 2, 3, and 4 involve divided visual attention, because the participant must attend to both the central and peripheral locations simultaneously. Tasks 3 and 4 additionally involve selective attention, because the participant must suppress processing of irrelevant distractors. Task 4 involves a more challenging selective attention task than Task 3, as the stimuli are similar to the distractors in Task 4. Thus, Tasks 2 and 4 differ in whether the participant must suppress processing of irrelevant target-like distractors. A system that is more efficient should spend fewer attentional resources processing irrelevant information, and thus a more efficient system should treat Tasks 2 and 4 more similarly.

Any type of training that is used to improve processing speed will most likely affect many cognitive domains such as attention, memory, and other executive function abilities (Ball et al., 2007). It is unknown if the improvements in attentional resource allocation observed here result directly from training, or are a by-product of having improved processing speed. Future work may dissociate these two possibilities. What these data *do* show is that participants are more efficiently allocating attentional resources after PST. After exposure to the modified version of PST, the initially robust task-related differences in pupil diameter decreased so that pupil diameter was indistinguishable between Tasks 2 and 4. Interpreting pupil diameter as a measure of attentional resource allocation, these data indicate that after training, the same level of attentional resources is allocated for both the difficult and easy tasks. This is consistent with the hypothesis that participants are more efficiently allocating attentional resources after PST.

This improvement in efficiency could allow for handling of more difficult visual tasks, or allow for multiple attentional processes to be completed without taxing the available attentional resources. The modified PST paradigm used was modeled after an established protocol that has been shown to transfer to a wide range of everyday activities in older adults including better health, maintained mobility, and improved driving safety (Edwards et al., 2009a,b; Ball et al., 2010; Wolinsky et al., 2010). Given that PST impacts a wide range of outcomes, it is likely that this training influences a factor that is important for a wide range of cognitive tasks. Allocation of attentional resources is one such factor. Our results are consistent with the hypothesis that cognitive training involves development of more efficient use of the available attentional resources.

This hypothesis also aligns with cognitive aging literature, as a range of studies have shown that some cognitive deficits in older adults may arise from their less efficient allocation of attentional resources (Gazzaley and D'Esposito, 2007; Vaden et al., 2012). Problems with attentional modulation cause a host of difficulties in a range of activities. For example, deficits in the ability to filter out distracting information can lead to poor performance on visual tasks (May et al., 1999; Yotsumoto and Sekuler, 2006). Improvements in the efficiency of the attention system may ameliorate these visual processing problems, allowing a participant to spend fewer attentional resources on a visual attention task.

These data might represent a mechanism in younger adults that underlies the benefits of diverse types of training, including both action video game training and PST. The presented findings are consistent with recent imaging studies examining the neural basis for the improvements seen through action video game training. Compared to non-gamers, action video game players did not activate the frontal-parietal attention network as attentional demands increased (Bavelier et al., 2011). Their results suggest that, following video game training, fewer attentional resources were required for more difficult tasks. Although our study used different methodology, a similar conclusion links the two different training paradigms.

There are some limitations to this study. First, although we are able to show that some physiological changes (pupillometry) occur as a result of training, we are not able to determine the training mechanisms underlying the behavioral transfer effects often found in the literature. This is likely due to two main issues. First, while the sample size is reasonable (based on the other literature) for pupillometry, it is very small for the detection of transfer to behavioral tasks. Other PST research, which found near and far transfer effects in older adults, included samples from 97 to over several thousand participants (for a review, see Ball et al., 2007). Next, and perhaps more importantly, the modified UFOV test has a limited range and a strong ceiling effect (Edwards et al., 2006) that can be hit even in the older adult population. As such, this ceiling was more extreme in our healthy younger adult sample whose fluid abilities and processing speed are at their best functioning during the life course (Schaie, 1996). The UFOV would need to be further altered to be made more difficult to be able to detect behavioral differences in this sample. Making such modifications would have further removed this previously standardized, valid and reliable measure away from the established literature. Thus, we decided to make as few modifications as possible for use in the younger adult population. Finally, another limitation is that our pupil diameter measurement is relative within a session. Pupil diameters are measured with respect to a within-session baseline. Therefore, all the measures of interest are changes in pupil diameter within a session, rather than raw pupil diameter (See Figure 2). Thus, the inferences we can make based on these data reflect task-driven changes in attentional allocation, rather than raw values. Despite these limitations, our data still reveal important insights regarding objective changes in attentional resources as a result of training.

PST has been extensively studied in an older adults, and has shown to have the strongest behavioral benefits in those participants who have some baseline processing speed difficulties (Ball et al., 2007). Although the younger adults used in this study did not have the same baseline processing speed difficulties, gains in efficiency were still found, indicating that improvements might be made even before the onset of age-related declines. This idea is reflected in the action video game literature where young healthy adults improve in many aspects of attention after gaming.

## Conclusion and future directions

Here we studied one possible basic mechanism involved in PST in a group of healthy young adults, a group that is understudied in the current PST literature. Our data are consistent with the hypothesis that PST increases the efficiency of attentional resource allocation. Examining training effects in a healthy young adult population allows us to understand training mechanisms independently from the complicating effects of aging. Future work should examine these mechanisms in an older adult group. Work should also address whether the pupil diameter effects we observe are related to the degree of transfer to performance in everyday activities.

### Conflict of interest statement

The authors declare that the research was conducted in the absence of any commercial or financial relationships that could be construed as a potential conflict of interest.

## Footnotes

^1^Copyright © 1999 Visual Awareness Research Group, Inc., Chicago, Illinois, U.S.A. All rights reserved.
